# Implementation of a Multidisciplinary Protocol for the Safe Handling of Iodine‐125 Radioactive Seeds in the Pathology Laboratory

**DOI:** 10.1155/ijbc/3962099

**Published:** 2026-05-12

**Authors:** Amanda Rodríguez-Villena, Ute Corina Vera Schmülling, Irene Vicente Zapata, Sonia Rivas-Fidalgo, María Gión-Cortés, María Jesús López-Rodríguez, Irene Carretero-Barrio, José Palacios, Belén Pérez-Mies

**Affiliations:** ^1^ Department of Pathology, Ramón y Cajal University Hospital, Ramón y Cajal Health Research Institute (IRYCIS), Madrid, Spain; ^2^ Faculty of Medicine, University of Alcalá, Madrid, Spain, uah.es; ^3^ Department of Nuclear Medicine, Ramón y Cajal University Hospital, Ramón y Cajal Health Research Institute (IRYCIS), Madrid, Spain; ^4^ Department of Radiology, Ramón y Cajal University Hospital, Ramón y Cajal Health Research Institute (IRYCIS), Madrid, Spain; ^5^ Department of General Surgery, Ramón y Cajal University Hospital, Ramón y Cajal Health Research Institute (IRYCIS), Madrid, Spain; ^6^ Department of Medical Oncology, Ramón y Cajal University Hospital, Ramón y Cajal Health Research Institute (IRYCIS), Madrid, Spain; ^7^ Department of Obstetrics and Gynecology, Ramón y Cajal University Hospital, Ramón y Cajal Health Research Institute (IRYCIS), Madrid, Spain; ^8^ CIBERONC, Instituto de Salud Carlos III, Madrid, Spain, isciii.es

## Abstract

**Background:**

Iodine‐125 radioactive seeds (I_125_‐RS) are increasingly used for preoperative localization of nonpalpable breast lesions due to their precision, safety, and logistical advantages. However, due to radiation safety regulations, their use requires a coordinated and strictly regulated workflow to ensure safe handling and retrieval of radioactive material. This study assesses the first year of a multidisciplinary protocol implemented to standardize I_125_‐RS management and minimize incidents.

**Methods:**

A protocol involving Radiology, Surgery, Nuclear Medicine, and Pathology was established to ensure proper documentation, transport, radiographic confirmation, seed retrieval, and incident reporting. All I_125_‐RS recovered from formalin‐fixed specimens between May 2022 and September 2023 were evaluated. Incidents were classified as protocol deviations (Category I) or technical retrieval difficulties (Category II). Clinicopathological data were collected, and analyses were performed using chi‐square and ANOVA tests (*p* < 0.05).

**Results:**

A total of 146 seeds were retrieved from 130 specimens from 123 patients. Most samples were lumpectomies (89.2%); 24.4% of patients received neoadjuvant therapy. Invasive carcinoma accounted for 82.3% of diagnoses. Double‐seed placement was used in 16 cases. Positive margins occurred in 9.1%, and 7.2% required reoperation. There were 18 incidents (14.6%): Category I, 10 (55.6%), mainly documentation errors or seed displacement within containers; and Category II, 8 (44.4%), mostly seeds hidden within tissue slices. Nuclear Medicine was consulted in 11 cases. Importantly, no seeds were lost. No significant associations were found between incidents and clinicopathological variables.

**Conclusions:**

The multidisciplinary protocol ensured safe and efficient handling of I_125_‐RS, preventing seed loss and improving traceability. Most incidents were minor, manageable, and reduced with staff training and adherence to standardized procedures. Continuous monitoring supports workflow optimization and maintains safety standards in pathology laboratories. This approach may serve as a model for institutions implementing I_125_‐RS localization.

## 1. Background

The number of nonpalpable breast lesions requiring presurgical localization has increased, driven by population aging, expanded mammographic screening, and greater use of neoadjuvant therapy (NAT) [1]. Although hook‐wire localization (HWL) has traditionally been the standard, its limitations have led to the adoption of less invasive and more flexible alternatives. Among these, iodine‐125 radioactive seed (I_125_‐RS) localization is the most widely used, being also applied to mark axillary lymph nodes, particularly in NAT [2].

In this technique, a small titanium seed (4.5 mm) containing a low dose of I_125_ is placed in the nonpalpable breast lesion or lymph node under image guidance by a radiologist [1–3]. During surgery, the seed is identified with a gamma probe and later retrieved in the Pathology Department. I_125_‐RS is considered safe and requires no protective measures beyond standard practice [4, 5]. Miodownik et al. [4] reported dose optimization to 2.8 MBq under ALARA principles, eliminating the need for personnel dose monitoring. Pluim et al. [6] recently confirmed fetal safety during pregnancy.

I_125_‐RS are reusable, and compatible with magnetic resonance imaging (MRI) (unlike Magseed) and electrocautery (unlike SAVI Scout) [3]. Gamma probes detect their 27‐keV emissions [1, 2] without diffusion artifacts or interference with Tc99 sentinel node detection [2, 7, 8], reaching depths greater than 10 cm [9]. However, their placement is not recommended in cystic or hemorrhagic lesions due to the risk of migration or loss [3, 4].

At our institution, I_125_‐RS is the preferred method for presurgical localization of nonpalpable lesions. While many centers are considering its implementation, concerns about handling radioactive material persist, despite its compliance with national safety regulations [4]. In Spain, for example, loss of any radioactive seed must be reported to the Nuclear Safety Council [9, 10].

During the initial implementation, some difficulties in seed retrieval were observed, mainly due to staff turnover and unawareness of the recovery procedures, as well as occasional seed detachment during specimen radiography or surgery. These issues underscored the need for a multidisciplinary protocol ensuring proper localization, retrieval, and traceability, which led to the design of the present standardized workflow.

Most publications focusing on I_125_‐RS address surgical outcomes, whereas limited data exist on seed handling within the pathology laboratory. This study evaluates the implementation of the I_125_‐RS handling protocol during its first year and compares the results with data reported in the literature to assess its effectiveness and safety.

## 2. Materials and Methods

### 2.1. Protocol Development

**Figure 1:**
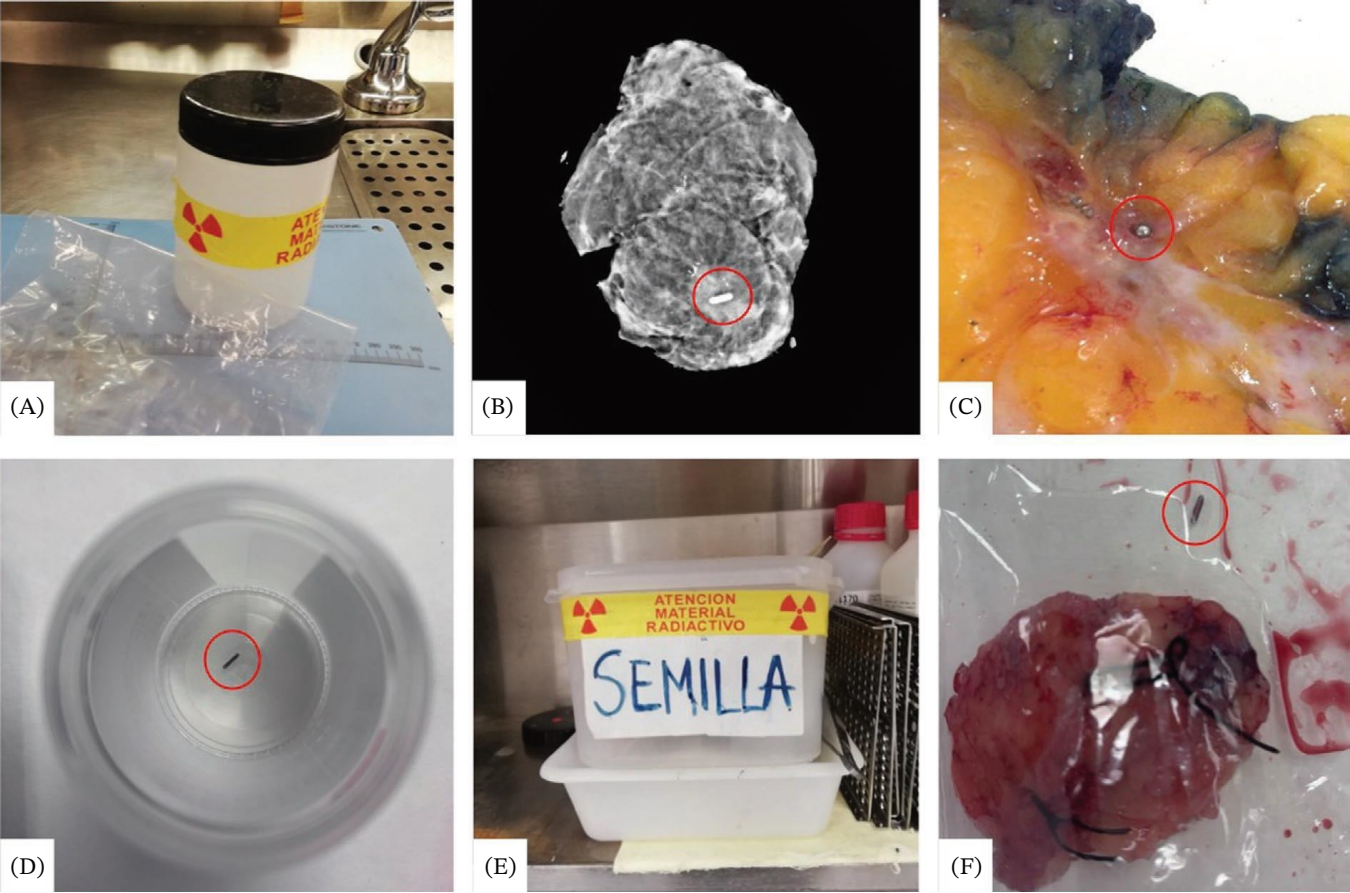
Management of specimens containing I_125_‐RS in the Pathology Department. All I_125_‐RS are encircled in red. (A) Double container (plastic bag within a radiation‐labeled container). (B) Radiographic confirmation. I_125_‐RS is inside the specimen. (C) Identification and extraction of I_125_‐RS: gross sectioning of a specimen also marked by a hydrogel coil. A hemorrhagic tissue reaction surrounding the seed can be observed. (D) Storage of extracted seeds in individually labeled containers. (E) Radiation‐labeled container used to store all seeds retrieved during the day until collected by Nuclear Medicine. (F) Incident example: I_125_‐RS had migrated out of the surgical specimen but remained inside the specimen bag, preventing loss.

A multidisciplinary protocol involving the Radiology, Surgery, Nuclear Medicine, and Pathology departments was developed to minimize the risk of seed loss. Before sending specimens to Pathology, the surgical team specifies the number of seeds and seals the specimen in a radiation‐labeled container, and the seed position is confirmed radiographically. Pathologists verify documentation, locate and remove the seed, store it in a radiation‐labeled container, and record the procedure in the gross report. If a seed cannot be located, Nuclear Medicine is contacted immediately, and no material is discarded until retrieval (see Table 1 and Figure 1).

**Table 1:** Detailed multidisciplinary protocol for handling I_125_‐RS–labeled specimens.

**Seed retrieval multidisciplinary protocol**
Step 1. Radiology and surgical teams • *Seed placement and specimen handling:* The radiologist places the I_125_‐RS in the target lesion under image guidance. • After excision, *the surgeon seals the specimen* in a plastic bag, places it in a rigid, *radiation-labeled container*, and records the number of seeds on the pathology form. • *Radiographic confirmation:* The presence and position of the seed(s) are verified by intraoperative radiography without opening the sealed bag to avoid seed loss.
Step 2. Reception in the Pathology Department • *Communication with the Nuclear Medicine Department:* Each week, Nuclear Medicine staff informs the Pathology Department of scheduled seed localization cases and provides prelabeled tags for proper seed identification and tracking. • *Verification of documentation:* Check that labeling and documentation are complete and consistent with the surgical report and that the specimen complies with the safety protocol. • *Retention of packaging:* Keep all packaging materials secure until seed removal has been completed and confirmed.
Step 3. Macroscopic processing • *Seed identification and removal:* Locate the radioactive seed within the specimen and carefully remove it to prevent rupture or displacement. Make parallel thin sections (2–3 mm thick) to extract the seed while preserving the specimen for analysis. Avoid using scissors or instruments that may damage or rupture the seed. • *Seed storage:* Place the recovered seed in a secure, clearly labeled container with patient identifiers and a radioactive warning. • *Documentation:* Record the presence, handling, and storage of the seed in the gross report. • *Seed retrieval:* The container is stored in the laboratory until collection by the Nuclear Medicine team. The seed is then transferred to the Nuclear Medicine Department for recording and processing according to institutional radioactive material handling protocols.
Step 4. Incident management • *Unlocated seed in the Pathology Department:* Contact the Nuclear Medicine Department immediately. Do not discard any specimen material or instruments until it is secured. • *Displaced seed prior to its arrival at the Pathology Department:* If the seed detaches from the specimen before reaching the Pathology Department, the responsible clinician must recover it, inform the Nuclear Medicine Department, and note the incident in the surgical report. Nuclear Medicine staff will confirm recovery. • *Documentation:* All incidents must be recorded for review, classification, and evaluation to monitor protocol compliance.

### 2.2. Recording and Classification of Incidents

All incidents were recorded and subsequently classified by our team as either protocol deviations (Category I) or technical retrieval difficulties (Category II). Calls to the Nuclear Medicine Department were also recorded. Before implementation, staff received training on the protocol and incident management.

### 2.3. Study Design and Statistical Analysis

To assess protocol performance, all I_125_‐RS retrieved from breast and axillary formalin‐fixed specimens between May 2022 and September 2023 were analyzed. Intraoperative specimens were not included in the study. Collected data included patient age, specimen type and size, NAT status, histological subtype, margin involvement (only lumpectomies with malignant histology), number of seeds, and recorded incidents.

Statistical analyses were performed using SPSS v25.0 (IBM Corp., Armonk, New York, United States). Associations between incidents and clinicopathological variables were assessed using chi‐square or ANOVA tests, with significance set at *p* < 0.05.

## 3. Results

A total of 146 I_125_‐RS were retrieved from 130 specimens from 123 patients. Most specimens were lumpectomies (116/130, 89.2%), and 14 were sentinel lymph nodes (10.8%). Thirty patients (24.4%) had received NAT.

The mean patient age was 58.1 years (range 31–86), and the mean specimen size was 4.5 cm (range 1–18). Histologically, 107 cases (82.3%) were invasive carcinoma, 17 (13.1%) ductal carcinomas in situ (DCIS), and 6 (4.6%) other lesions (angiosarcoma, intraductal papilloma, phyllodes tumor, or B3 lesions). Double‐seed placement was performed in 16 cases, primarily in multifocal invasive carcinomas (43.8%), post‐neoadjuvant cases (25%), DCIS (25%), and one papilloma (6.3%). Of the 111 lumpectomies evaluated, 10 (9.1%) had positive margins, and 8 (7.2%) required reoperation.

Incidents occurred in 18 patients (14.6%): 10 protocol deviations (Category I) and 8 seed localization difficulties (Category II) (Table 2). Nuclear Medicine was contacted in 11 cases (8.9%), predominantly Category II incidents (8/11). None of the 146 seeds were lost.

**Table 2:** Incidents documented during the handling of I_125_‐RS–labeled specimens in the pathology laboratory.

	*N*	%
Category I: Noncompliance with the protocol	10	56
I_125_‐RS inside the container, out of the specimen	4	22.2
Presence of I_125_‐RS not indicated in the request	3	16.6
Wrong number of I_125_‐RS indicated in the request	1	5.5
Absence of a radioactive material sticker on the container	1	5.5
I_125_‐RS found inside the patient, out of the specimen	1	5.5
Category II: Difficulties during seed retrieval	8	44
Parallel cuts along the seed path (I_125_‐RS, hidden within a tissue slice)	6	33.3
I_125_‐RS inside the specimen, outside the tumor	1	5.5
Confusion with coil	1	5.5
Total incidents	18	100

No significant associations were observed between the occurrence of incidents and clinicopathological variables, including patient age (ANOVA, *F* = 0.21, *p* = 0.645), specimen size (ANOVA, *F* = 0.36, *p* = 0.547), NAT status (*χ* ^2^ = 1.57, *p* = 0.210), specimen type (*χ* ^2^ = 2.25, *p* = 0.134), or the use of multiple seeds (*χ* ^2^ = 0.01, *p* = 0.904). However, incidents were strongly associated with the need to contact the Nuclear Medicine Department (*χ* ^2^ = 26.1, *p* < 0.001). Incidents were more frequent in sentinel lymph node specimens than in lumpectomies (28.6% vs. 12.1%), although this difference did not reach statistical significance (*χ* ^2^ = 3.18, *p* = 0.075).

## 4. Discussion

Despite the strict Nuclear Safety Regulations required for their use, I_125_‐RS are currently the most widely adopted tumor localization method [3–5]. Their very low radioactivity (< 4 MBq) [7] ensures safety for both patients and healthcare staff [3–5]. Compared to HWL, I_125_‐RS provide greater surgical flexibility, as they can be implanted up to several days or weeks in advance of surgery. Published series describe safe in situ intervals ranging from several days to multiple weeks [1–5], allowing real‐time intraoperative adjustments [11] and making them suitable for use in patients undergoing NAT due to their 59.5‐day half‐life [10]. Besides, lower anxiety levels and better cosmetic outcomes have been reported [3]. I_125_‐RS also offer superior precision, with a minimal migration risk (< 1%) [8, 11], preserving histological integrity [7].

Patient demographics and histological types in our study were consistent with previous literature [2, 4, 8]. Sánchez et al. [8] observed smaller specimen sizes with I_125_‐RS than with HWL (4.9 cm vs. 5.41 cm), consistent with our findings (4.5 cm). Similarly, in our cohort, larger or multifocal specimens, post‐NAT tumor beds, and ill‐defined lesions (e.g., papillary neoplasms) more often required two seeds [2, 3, 5, 11].

Rates of positive margins and reoperations with I_125_‐RS are noninferior or superior to HWL and nonradioactive seed (No‐RS) [1–3, 8, 12]. In our study, positive margins occurred in 9.1% of lumpectomies, with a reoperation rate of 7.2%, lower than Taylor et al.’s [2] rate (13.9%), likely due to a smaller proportion of DCIS. Janssen et al. [13] reported that using two seeds in extensive DCIS lesions (> 3 cm) reduced reoperation rates (39.6% with one seed vs. 20.7% with two). In our series, none of the DCIS cases marked with two seeds required reoperation. Liang et al. [3] reported positive margin rates of 9% for HWL and 11% for both I_125_‐RS and No‐RS (*p* = 0.4), slightly higher than ours (9.1%). Sánchez et al. [8] also observed marginally higher rates of positive margins (11.6%) and reoperation (9.5%). Comparisons across studies are limited by variability in margin assessment criteria [2].

In patients undergoing NAT, I_125_‐RS have two main advantages: precise delineation of the tumor bed prior to treatment [5] and MRI compatibility for posttreatment response assessment [3, 9]. In our series, 20.7% of lumpectomies followed NAT, with 87% achieving clear margins and 8.7% requiring reoperation. These results are comparable to Rebollo et al. [10] (92.1%) and align with Janssen et al. [13], who reported the lowest rate of positive margins (80.9%). These findings support I_125_‐RS as a reliable localization method after NAT.

The most frequent incidents were related to seed traceability or displacement outside the specimen (Category I, 55.6%), which are reversible following standardized handling protocols. Category II incidents, related to seed identification within the specimen, result from parallel cuts through the seed track, leaving the seed embedded within a tissue slice where it is not easily identified. In these cases, the seed insertion trace or tissue reaction can provide guidance. In our experience, the frequency of such incidents decreases with increasing pathologist experience.

Previous studies have reported complications such as I_125_‐RS displacement or seed transection [2, 3, 5], which can be minimized by sectioning fixed tissue in thin, parallel slices, as recommended by Graham et al. [14]. Seed fracture is rare. In our experience, only one seed has ever fractured in a defective device, and this occurred outside the present study. Its occurrence is rare with the use of standard instruments but possible with a microtome, highlighting the need for recovery protocols [7, 14]. Consistent with the literature, no seeds were lost [14]. Nevertheless, it remains important to include procedures for managing lost seeds in the handling protocols.

Eleven of 18 incidents required nuclear medicine intervention, most of them Category II. Availability of a gamma detector within the pathology laboratory could streamline intraoperative assessments [3, 5, 14].

Economically, I_125_‐RS have proven more cost‐effective than other localization methods. Law et al. [1] demonstrated long‐term savings through reduced material and labor costs. However, institutions lacking nuclear medicine facilities face substantial initial investment in licensing, training, and regulatory compliance [1, 5]. In such cases, No‐RS may represent a practical alternative [3, 5, 15].

A limitation of this study is the absence of intraoperative specimen analysis, as only formalin‐fixed specimens were included. Fresh breast tissue is difficult to handle and often requires additional consultations with the Nuclear Medicine Department.

## 5. Conclusions

Successful implementation of a protocol for I_125_‐RS management requires a multidisciplinary approach and adherence to standardized handling protocols, ensuring optimal outcomes for both patients and clinical teams. Recording incidents and monitoring adherence are essential components for maintaining safety and optimizing workflow efficiency [12].

**Nomenclature**
I_125_‐RS: iodine‐125 radioactive seed
NAT: neoadjuvant therapy
HWL: hook‐wire localization
MRI: magnetic resonance imaging
No‐RS: nonradioactive seed

## Author Contributions

All authors contributed to the study conception and design. Material preparation, data collection, and analysis were performed by Amanda Rodríguez‐Villena and Belén Pérez‐Mies. The first draft of the manuscript was written by Amanda Rodríguez‐Villena, Irene Carretero‐Barrio, and Belén Pérez‐Mies, and all authors commented on previous versions of the manuscript.

## Funding

This study has been funded by the Instituto de Salud Carlos III (ISCIII) (through projects “PI22/01892” and “PI25/01933”), by CIBERONC (grant CB16/12/00316), and by the European Development Regional Fund “A way to achieve Europe” (FEDER).

## Disclosure

All authors read and approved the final manuscript.

## Ethics Statement

This study was approved by the research ethics board at our institution (reference number 049/24).

## Conflicts of Interest

The authors declare no conflicts of interest.

## Data Availability

All data generated or analyzed during this study are included in this published article.
